# Oxygen-induced cell migration and on-line monitoring biomarkers modulation of cervical cancers on a microfluidic system

**DOI:** 10.1038/srep09643

**Published:** 2015-04-23

**Authors:** Xuexia Lin, Qiushui Chen, Wu Liu, Jie Zhang, Shiqi Wang, Zhixiong Lin, Jin-Ming Lin

**Affiliations:** 1 Beijing Key Laboratory of Microanalytical Methods and Instrumentation, Department of Chemistry, Tsinghua University, Beijing 100084, China; 2 The Key Laboratory of Bioorganic Phosphorus Chemistry & Chemical Biology, Department of Chemistry, Tsinghua University, Beijing 100084, China; 3 School of Science, Beijing University of Chemical Technology, Beijing 100029, China; 4 Department of Neurosurgery, First Affiliated Hospital of Fujian Medical University, Fuzhou, Fujian 350005, China

## Abstract

In this work, we report an integrated microfluidic device for cell co-culture under different concentrations of oxygen, in which the secreted protein VEGF_165_ was on-line qualitatively and semi-quantitatively analyzed by functional nucleic acid, hemin, ABTS and peroxide system. This microfluidic platform allowed investigation of various oxygen and distances effect on cell-to-cell communication. Besides, the microfluidic device was used for real-time analysis of VEGF_165_ protein by aptamer-functionalized microchannels. Under 5% O_2_ condition, we found that the migration of CaSki cells was faster than the migration of human umbilical vein endothelial cells. However, the migration of CaSki cells was slower than the migration of HUVECs under 15% O_2_ condition. Moreover, the shorter intercellular distances, the quicker cells migration. Furthermore, HIF-1α and VEGF_165_ genes, ROS were analyzed, and the results would provide new perspectives for the diagnosis and medical treatment of cervical cancer.

Oxygen level plays an important role in tumor growth, invasion and metastasis^1^,^2^. Low level of oxygen (hypoxia, <1%) induces tumor cell invasion into neighboring healthy vasculatures, resulting in metastasis^3^,^4^. Great efforts have been devoted into understanding the effects of oxygen level on tumor development *in vivo*. However, it is still difficult to study tumor development under different oxygen conditions because it is technically challenging in precisely controlling the conditions *in vivo* and *in vitro*, as well as monitoring cell-cell communication in the microenvironments. Although hypoxic culture chamber system is usually applied, it remains a challenge to mimic the *in-vivo* cellular microenvironment under biophysical stimuli such as the biomolecular transport gradient.

Microfluidic device has been increasingly emerging as a suitable platform for *in-vitro* mimicking oxygen gradient microenvironment because it regulates critical elements such as diffusion distance, and precisely controls the cellular and non-cellular microenvironment especially oxygen condition at the micrometer scale^5^,^6^,^7^. Previous works have been reported that different intercellular distances greatly affected on substance exchange and cell-cell communication^8^,^9^. In addition, oxygen gradients are generated in the microfluidic by using a flowing condition of pre-defined gas mixtures through channels^10^,^11^. However, there are few studies for different oxygen concentrations affected cell-cell interaction with real-time detection of cell secretions, which could provide insight into the tumor development. A microfluidic system has been designed to co-culture two types of cell in microchannels with channel altitude difference to promote nutrition and material exchange^12^,^13^. On-line analysis of cell co-culture metabolites is still challenged for in-situ monitoring biomarkers. An alternative strategy is to use aptamers for specifically capture of cell secreted vascular endothelial growth factor 165 (VEGF_165_)^14^,^15^,^16^. The captured proteins can be analyzed by functional nucleic acids with G-quadruplex DNAzyme, hemin, ABTS and peroxide system, which produces differences of color^17^,^18^. Thus, it can be analyzed semi-quantitatively by naked eyes without specialized instruments.

Herein, we presented a feasible investigation of the effects of various oxygen and distances on cell migration and cell communication by designing a two-layered microfluidic system. We presumed that under different oxygen contents, the amount of VEGF_165_ protein and ROS would be affected, and then influenced cellular behaviors (Fig. 1A). To prove this concept, CaSki cells (derived from cervical cancer) and human umbilical vein endothelial cells (HUVECs) were co-cultured in the microchannels as models of tumor cells (TCs) and endothelial cells (ECs), respectively (Fig. 1A). Under 5% O_2_ conditions, the migration of CaSki cells was faster than human umbilical vein endothelial cells, which might be a reflection of tumor invasion or tumor metastasis in cervical cancer. In contrast, the migration of CaSki cells was slower than HUVECs under 15% O_2_ conditions, which would promote angiogenesis. Moreover, the shorter intercellular distances, the quicker cells migration. To demonstrate the cell-cell interactions, the on-line analysis of VEGF_165_ (protein) was successfully achieved (Fig. 1). Furthermore, HIF-1α and VEGF165 genes, ROS were analyzed, and the results may provide deeper insights into tumor development^19^,^20^,^21^.

## Results

### Fabrication of two-layered microfluidic device

**Figure 1:**
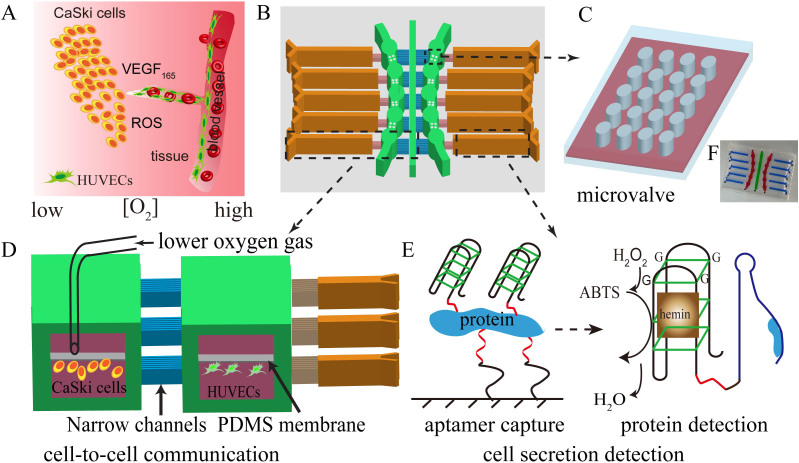
An integrated microfluidic device for cell co-culture under oxygen gradient system, in which for determination of the secreted protein VEGF_165_. (A) Oxygen effects cell-cell communication and promotes cell migration. (B) Schematic diagrams of the microfluidic device to mimic oxygen gradient and to observe cells migration. (C) The microvalve prepared by micro columns. (D) Two-layer microfluidic device for cells co-culture under low oxygen conditions. (E) Schematic illustration for determination VEGF_165_ based on nucleic acid aptamer. (F) The actual microfluidic device.

Two-layered microfluidic devices were designed with three various distances of channels (Fig. 1B, F). The cell culture chambers were 2.4 mm in diameter and 1.6 mm in width (Fig. 1B). The TCs was spatially cultured into the central microchannels (width 1.6 mm) and the cell-cell interactions were studied by using three different distances of narrow channels (Fig. 1D). The distance between three different chambers and the central channel were designed as 1.50 mm, 2.00 mm, 3.00 mm, respectively. The microchannels with 58 μm altitude differences were designed to control the cell growth microenvironment (Fig. 1D and Fig. S1 in Supporting Information). These altitude differences could prevent the cells entering into next channels and also allow the exchange of cellular soluble factors (see Fig. S2)^21^. PDMS membrane (20-μm thickness) has an excellent gas diffusion that was used to reduce the shear-stress produced by mixture gas (Fig. 1D and Fig. S1C)^5^,^22^. The outlet pressure changed slightly compared to the inlet pressure after applying the PDMS membrane in Fig. S3. We have also evaluated actual oxygen concentration in culture medium by applying Clark oxygen electrode. The results in Fig. S4 showed that the content of oxygen was changed. The shorter distance will lead to larger changes. The PDMS micro-columns were served as microvalves enabled to control the fluidic direction in virtue of its elastic characteristic (Fig. 1C, Fig. S1B and Supplementary Video 1). When the cell secretions were detected, the microchip was overturned. The liquid reflow can be greatly reduced when the microvalves were closed by mechanical force because some liquid was depressed to the low layer channel (Supplementary Video 2). More details of microfluidic device were described and discussed in the supporting information.

**Figure 2:**
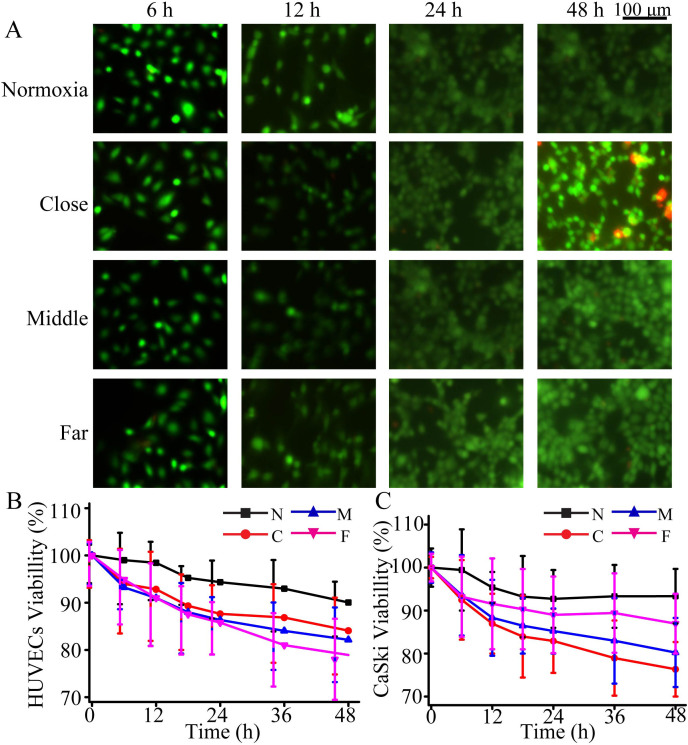
CaSki cells co-cultured with HUVECs under 5% O_2_ condition. (A) Fluorescent images of cells viability with HUVECs; (B) The viability of HUVECs; (C) The viability of CaSki cells. N indicated that cells under 21% O_2_ condition. C, M and F indicated that cells under 5% O_2_ condition with close, middle, and far distance between two cell lines, respectively.

CaSki cells and HUVECs were co-cultured in the microfluidic device to mimic the cellular microenvironment. Specially, CaSki cells were cultured under a mixture gas (5% O_2_, and 95% N_2_), while HUVECs were cultured under 21% O_2_ condition (21% O_2_ and 79% N_2_). 21% O_2_ conditions was set as a control because cells were cultured in 21% O_2_ conditions, there is little even no changes will be happen for cells in and out of the incubator. Control experiments were performed with the same condition except that both two types of cells were co-cultured in 21% O_2_ condition. The cell viability was accessed by cell live/dead kit under 5% O_2_. Fig. 2 (B–C) shows that cell viability was decreased with increasing period within 2 days. After 2 days, the viability of HUVECs and CaSki cells were still up to 80% and 86%, respectively. These results indicated that this microfluidic device can be suitable for cell-to-cell communication.

### Study of cell migration under 5% O_2_ condition

**Figure 3:**
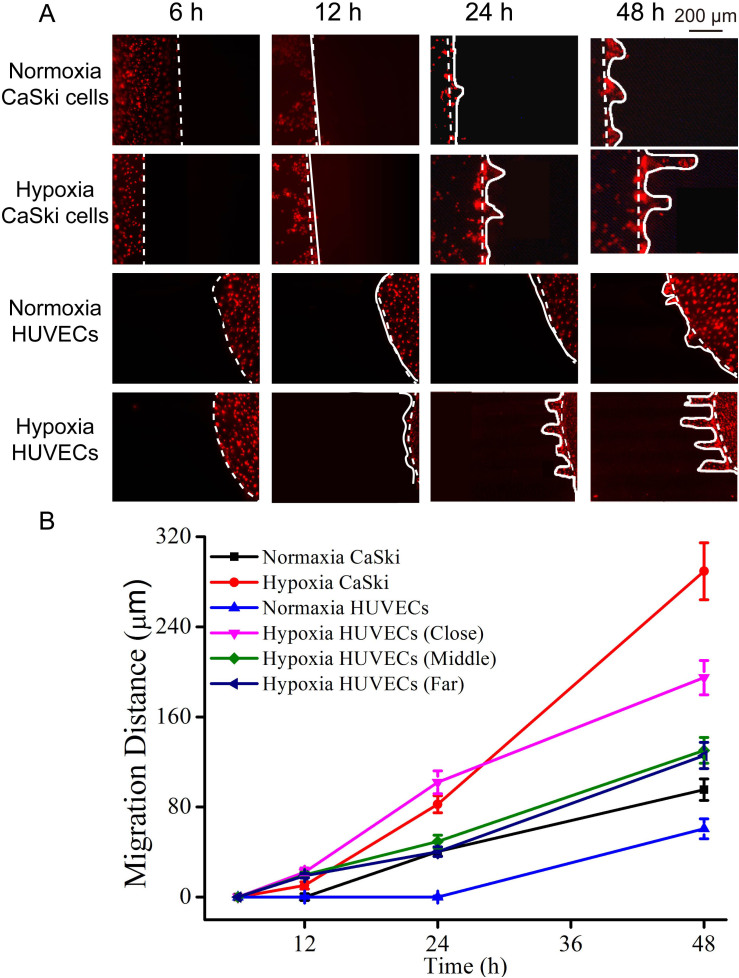
Cells migration under co-culturing with 5% O_2_ condition during 2 days. (A) Fluorescent images of cells migration. (B) The migration distance of CaSki cells and HUVECs in different chambers. Dashed line represents the cell positions at 0 h; the solid line represents the migration positions of cells in the corresponding time. Both two cells were labeled Dio fluorescent dye before cell seeding. Error bar was calculated by triplicate experiments.

The cell migration of both cell lines was studied under 5% O_2_ condition. In order to evaluate cellular migration, cells were labeled Dio fluorescent dye before cell seeding. After cell adhesion for 6 h, the distance was set as zero. After that, we introduced the mixture gas (5% O_2_, 95% N_2_), and recorded cell migration every 12 h. Finally, we calculated the cellular migration according to the distance of cells movement and the culture period. As shown in Fig. 3(A–B), the migration distance was set as zero when the co-cultured cells were filled with mixture gas in the microfluidic device. After 12 h co-cultured, neither cell lines migrated. For 24 h co-culture, it was clearly observed that both the two cell lines migrated from the original channel to the narrow channel. The migration rate of CaSki cells was higher than that of HUVECs in the far and middle chambers, but lower than that of HUVECs in the close chamber. Interestingly, the CaSki cells migrated faster than HUVECs from 24 h to 48 h. After 48 h co-culture, the farthest migration distances for CaSki cells and HUVECs were (289 ± 22) μm and (194 ± 19) μm, respectively. The migration rate of CaSki cells was higher than that of HUVECs. In addition, both two types of cells under 5% O_2_ migrated faster than the cells under 21% O_2_. Furthermore, the closer distance between two cell lines led to farther migration distance for cells movement, indicating the distance was an important factor for cell communication.

### Cell migration under 15% O_2_ condition

**Figure 4:**
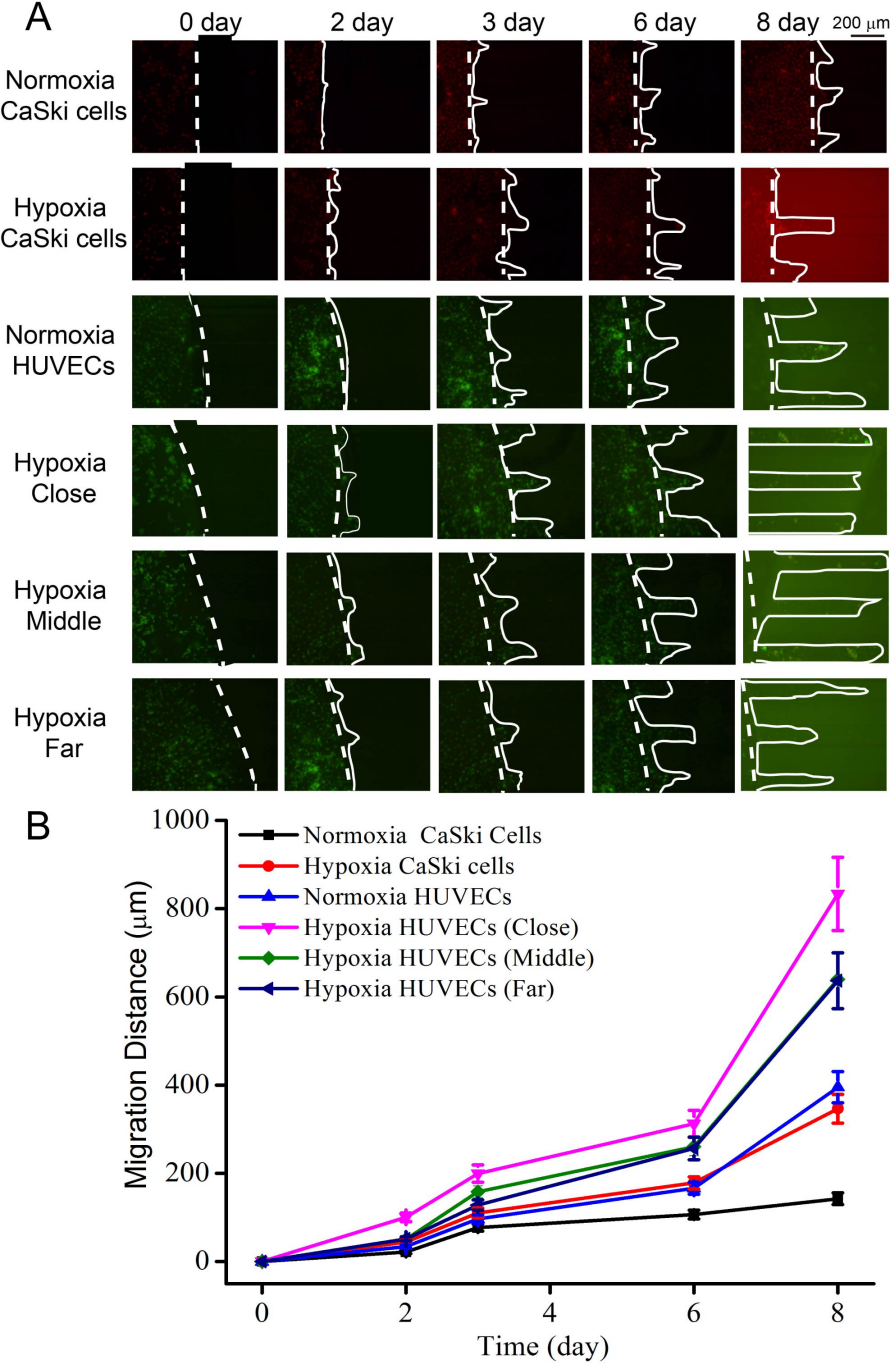
Cells migration under co-culturing at 15% O_2_ in 8 days. (A) Fluorescent images of Cells migration. (B) The migration distance of CaSki cells, and HUVECs in different chambers. CaSki cells and HUVECs were respectively labeled DiI and Dio fluorescent dye before cell seeding. Error bar was calculated by triplicate experiments. Dashed line represents the cell positions at 0 hr; the solid line represents the migration positions of cells in the corresponding time. Both two cells were labeled DiI fluorescent dye before cell seeding. Error bar was calculated by triplicate experiments.

To better understand the effects of oxygen level on cell-to-cell communication, CaSki cells and HUVECs were co-cultured in the microfluidic device under 15% O_2_ conditions. Choosing 15% oxygen as another level was due to some oxygen loss in the microchip and the level of oxygen in the arterial blood oxygen can be as high as 13%. To track cell movement, CaSki cells and HUVECs were respectively labeled with DiI and Dio dye before co-cultured on-chip. As shown in Fig. 4, after co-cultured 2 days, both two cell lines migrated. The migration rate has accelerated from day 3 and day 6. During day 6 to day 8, the migration rate of cells was greatly accelerated. After day 8, HUVEC cells were significantly migrated away from primary site, and the furthest migration distance was (833 ± 25) μm. In contrast, the CaSki cells only migrated about (350 ± 10) μm. Both two cell lines migrated faster under 15% O_2_ condition compared to under 21% O_2_ condition. Furthermore, the migration of CaSki cells was slower than HUVECs under 15% O_2_ condition. This was very different from under 5% O_2_ condition. Thus, the closer distance between two cell lines also resulted in higher cell migration ability.

### Study of HIF-1α and VEGF_165_ genes, VEGF_165_ protein, ROS under 5% and 15% O_2_ conditions

**Figure 5:**
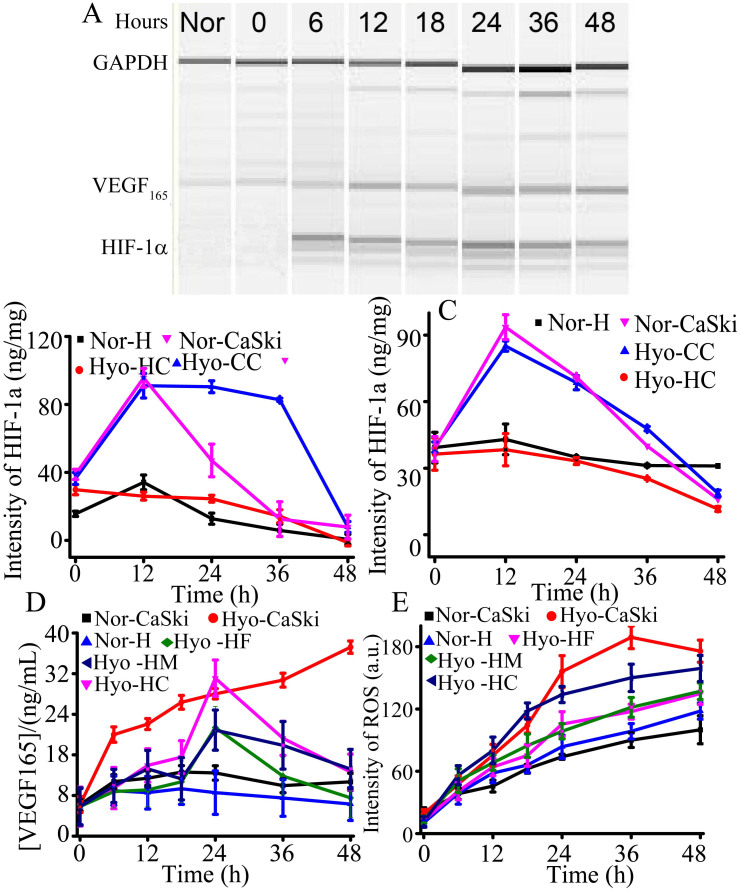
VEGF pathway during 48 hours at 5% O_2_. (A) Time-dependence HIF-1α and VEGF_165_ genes for CaSki cells by 2% hydroxyethyl cellulose electrophoresis. The control gene was GAPDH. (B)HIF-1α protein expression from the close chamber; (C)HIF-1α protein expression from the far chamber; (D) VEGF_165_ protein expression; (E) ROS intensity. Normoxia indicated that cells culture with 21% O_2_, the other cells cultured under 5% O_2_. Nor means both the cells co-culture under normoxic condition. Hyo means the cells co-culture under hypoxia condition. H indicates HUVECs. HF indicates HUVECs cultured in the far chamber. HC indicates HUVECs cultured in the close chamber. HM indicates HUVECs cultured in the middle chamber.

**Figure 6:**
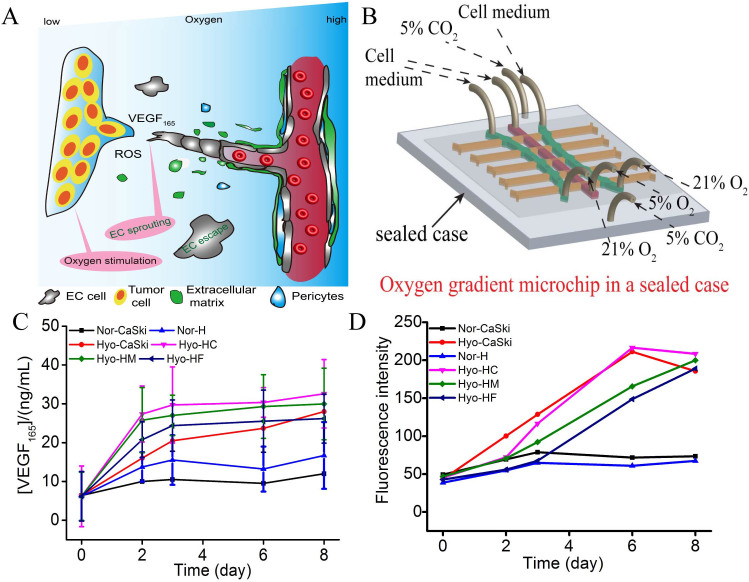
Cells stimulation and secretion at 15% O_2_. (A) The possible cell-to-cell communication mode between the CaSki cells and HUVECS model; (B) Oxygen gradient microchip in a sealed case; (C) VEGF_165_ protein expression; (D) ROS intensity.

HIF-1α, ROS and VEGF_165_ are three well-known biomarkers in tumor angiogenesis and tumor invasion. High level of VEGF_165_ protein enhances the cell permeability to accelerate the tumor cell migration^23^,^24^. High ROS generation might result in the slower cell migration because of cell damage, which could cause tumor invasion due to the relatively higher oxygen tension required at the invasive front^25^,^26^. To investigate the biological principle under the phenomenon, HIF-1α and VEGF_165_ genes were analyzed by the combination of RT-PCR and microchip electrophoresis. VEGF_165_ protein was on-line analyzed by functional nucleic acids consisting of aptamer and G-quadruplex structure in Fig. (S5–S6)^17^,^27^. The details of VEGF_165_ capture and detection were discussed in supporting information. As shown in Fig. 5A and Fig. S7, the HIF-1α gene was raised with time under 5% O_2_ condition, which indicated that HIF-1α was activated and accumulated under 5% O_2_ environment. HIF-1α protein was analyzed by the standard ELISA kit (Cloud-clone Corp, USA) and the total protein was analyzed by Bradford kit (BiomigaInc., USA). As shown in Fig. 5B, the content of HIF-1α protein was increased within 12 hours and the intensity remained at about 85 ng/mg from 12 h to 36 h, then was decreased when HUVEC cells was co-cultured in the close chamber during 48 h. The content of HIF-1α protein was increased within 12 h, and then gradually decreased when HUVEC cells was co-cultured in the far chamber during 48 h in Fig. 5C. These results indicated that the closer distance led to the lower oxygen, and then the lower concentration of oxygen can reduce HIF-1α degradation. These results were consistent of the oxygen gradient related to the distance in Fig. S4. Furthermore, the results also indicated that the distance effected cellular communication. Moreover, the VEGF_165_ gene was also increased depending on the time extension, indicating HIF-1α may up-regulate VEGF_165_ gene. As shown in Fig. 5D, the VEGF_165_ protein secreted by CaSki cells increased rapidly during 6 h, and then slowed down from 24 h to 48 h, indicating that 5% O_2_ stimulation enhanced the VEGF_165_ protein expression in TCs. In HUVECs, VEGF_165_ protein also increased with increasing time during the beginning 24 h, and then decreased from 24 h to 48 h. Besides, the shorter distance, the bigger changes of VEGF_165_ protein led to high migration activity of HUVECs in closer chamber. The expression of VEGF_165_ protein after 24 h in HUVECs was lower than that of CaSki cells. Under 15% O_2_ conditions, a number of VEGF_165_ were secreted during the first two days, and maintained from 2 days to 8 days co-culture in Fig. 6C. To ensure the reliable results, a VEGF Quantitative ELISA kit was used to analyze VEGF_165_ in cell medium under oxygen stimulation. The trends of VEGF_165_ protein between using ELISA Kit and the established method are in agreement.

ROS is usually contributed to various aspects of malignant tumors, including carcinogenesis, aberrant growth, metastasis, and angiogenesis^28^,^29^. Under a 21% O_2_ microenvironment, the generation of ROS maintained at a low frequency because it could easily be neutralized by antioxidant defenses in Fig. 5E. The content of ROS under 5% O_2_ was higher than that under 21% O_2_, indicating that the balance of antioxidant defenses might be broken. During the first 24 h, the generation of ROS in both cells was higher than that after 24 h, which is different for the cell migration. Fig. 6D shows ROS generated quickly in the primary 6 days under 15% O_2_. After 6 days, the concentration of ROS was also increased except that from CaSki cells and HUVECs in close chamber under hypoxic conditions. Besides, in the close distance, the content of ROS was high, demonstrating that the distance affected the ROS diffusion.

## Discussion

In this work, we have successfully developed a two-layered microfluidic system to study the effects of various oxygen and distances on cell migration. The effect of oxygen content for cell migration was investigated by introducing different oxygen of 5% O_2_ and 15% O_2_ respectively into the up-layer of the main microchannels. Besides, to keep the pH of culturing cell microenvironment was stable, a mixture gas with 5% CO_2_, 21% O_2_ and 74% N_2_ was incessantly provided (Supplementary Video 3 and Fig. 6B).

In order to get low content of oxygen, we firstly optimized the distance between microchannel and chambers to make sure the substance can be exchanged in time for cells. When the distance of microchannel and chambers was range from 1.20 mm to 3.00, the liquid can be reached adjacent channel and chambers during 30 min. If the larger distances were set, it was hard to exchange substance effectively. When the distances were lower than 1.20 mm, the substance was too earliest to exchange. The stability of oxygen content in the system was studied. When the main channel introduced hypoxia conditions (<1% O_2_), the oxygen content was increased to 4% during 2 days. Then, to keep the low oxygen content, we take the process by increasing the gas flow rate. Unfortunately, high flow rate enhanced the gas exchange, which is also hard to keep stability of oxygen content under the condition of 1% oxygen. Nevertheless, the system exhibited a good stability when the oxygen content was higher than 4%. Therefore, to mimic physiological growth conditions and to better understand the cell communication^31^,^32^,^33^, we exposed TCs and ECs to 5% O_2_ condition and the system as following: the lowest distance was set as 1.2 mm, and the low average distance was about 1.5 mm. Finally, we are still looking for new methods or materials for study oxygen effect especially the hypoxic (<1% O_2_) study.

VEGF has been shown to play an important role in angiogenesis of cardiovascular and cerebral ischemia, which can be overexpressed by activated HIF-1α. Hence, the expression of HIF-1α and VEGF165 were studied by inducing the different content of oxygen. Under 5% O_2_ condition, HIF-1α accumulated within the first 12 h, and the concentration merely changed from 12 h to 36 h in vicinity chamber (Fig. 5B). On the contrary, it rapidly decreased from 12 h to 36 h in relatively farther chamber (Fig. 5C). These results indicated the oxygen content effect HIF-1α expression during the 48 h. Then we applied a G-quadruplex, hemin, ABTS and peroxide system to detect VEGF_165_ protein. We observed obvious color change as a marker for different amount of cell secretion VEGF_165_ protein. Under 5% O_2_ conditions, VEGF_165_ increased in the closest TCs culture chamber, where cells also moved a farthest distance. For HUVECs, the concentration of VEGF_165_ under 5% O_2_ conditions was also higher than that under 21% O_2_ conditions, thus promoting a more active cell migration. Besides, a common rule was that shorter the distance between two types of cell culture chamber, the more VEGF_165_ cell would secrete. More VEGF_165_ resulted in farther migration, although after 24 h the concentration of VEGF_165_ decreased anyway. Furthermore, TCs secreted more VEGF_165_ than ECs under 5% O_2_, which explained why the migration of TCs was faster than ECs in the same time interval, although ECs themselves are very responsive to VEGF_165_. Since HUVECs are ECs, the higher viability of TCs may lead to faster movement than HUVECs.

High concentration of ROS is able to stimulate the generation of tumor, and could activate the expression of VEGF_165_, which accelerated CaSki cell migration in our experiment. Under 5% O_2_ conditions, the cell apoptosis was time-dependent and accelerated with the increasing period (Fig. S8 and Fig. S9). The apoptosis rate of CaSki cells was lower than HUVECs, indicating that the anti-hypoxia ability of HUVECs was inferior to CaSki cells. Besides, the cell apoptosis rate of HUVECs in vicinity chambers was higher than that in chambers of farther distance, which might be caused by cell damage from high-level ROS and VEGF_165_ protein^30^ induced by 5% O_2_ condition. These results revealed that the HUVECs presented a lower migration activity than CaSki cells. Cell proliferation was also studied in time-dependent growth pattern under 5% O_2_ condition (Fig. S10). During two days' co-culture, the number of CaSki cells and HUVECs both increased, and the CCK-8 absorbent became stronger. The growth rate of CaSki cells was about 1.25-fold that of HUVECs. The viability of CaSki cells was also higher than HUVECs (Fig. 2), illustrating that CaSki cells were more tolerant to low level of oxygen content than HUVECs. The higher viability and less inhibited proliferation in close co-culture of two cells made the CaSki cells move faster than HUVECs under 5% O_2_ condition, implying that the distance was an important factor for cell communication. In addition, high cell viability and proliferation rate under 21% O_2_ conditions indicated that this microfluidic system can be served to study the cell-cell communication under different oxygen content environments.

Under 15% O_2_ conditions, both types of cells migrated slowly within two days' co-culture. However, from Day 2 to Day 6, the migration rate was accelerated. The migration rate of cells was further accelerated from day 6 to day 8. Combination with cell secreting VEGF_165_ protein and ROS (Fig. 6), we found that when cells were cultured under 15% O_2_ condition, VEGF_165_ protein expressed at a stable level from day 2 to day 6. However, the ROS level was increased with the culture period, which might cause cell damage and lead to reduced VEGF_165_ expresion from day 6 to day 8. High ROS level also made HUVECs move slowly. During day 6 to day 8, the decline of ROS level in CaSki cells made HUVECs move faster to CaSki cell chamber. Besides, shorter distance between two cell lines can lead to faster migration rate of HUVECs, indicating that the distance was a significant influencing factor on cell migration. It was reasonable to interpret the above fact as that shorter distance accelerated substance exchange and promote cell migration. Thus, our strategy offered an excellent model for investigating the effect of oxygen level on cell migration, and might be potentially useful in predicting prognosis.

In summary, a two-layer microfluidic system was developed to study cell migration under two different low oxygen (5% and 15% O_2_) levels in vitro, and the cell secretion was monitored in real time. This system varied the distances for cell-cell co-culture, and allowed the substance exchanged by diffusion process. As important influencing factors in tumor development, ROS and VEGF_165_ were online detected in microfluidic device. The device was used to study cell-cell communication with different distances under 5% O_2_ and 15% O_2_ conditions. Hence, this study provides a better understanding of cervical cancer, which may introduce new perspectives for future therapy. Furthermore, the developed device could be employed for 3D cells co-culture, and we anticipate that the integrated device would not only be applied in investigating cell-cell communication, but also be used for therapeutic tumor or drug screening.

## Methods

### Fabrication of microfluidic device

A two-layer microfluidic device was fabricated using poly-dimethylsiloxane (PDMS, Sylgard 184, Dow Corning) by two steps of standard soft lithography and replicamolding techniques^12^,^13^. The top and bottom layers with microchannels were exactly the same. Briefly, negative photoresist SU-8 2050 (Microchem, Newton, MA) was spun onto a silica wafer (Tianjin, China) at a speed of 2500 rpm for 50.0 s using a spin-coater. After baking at 65.0°C for 10 min, UV light exposure and following development were done to generate the layer of narrow channels (24.0 μm thick). Then another layer of SU-8 2050 photoresist was coated onto the same wafer at a speed of 1000 rpm for 50.0 s. After baking at 65°C for 10.0 min, UV light exposure and development were done to generate the layer of cell culture chambers and main channels (82.0 μm heigh). 10.0 μL silylation reagents and the silica master were then put into vacuum kettle together to make the silica master hydrophobic. Then, a premixed 10:1 PDMS preploymer and curing agent (DowCorning, Sylgard 184, Midland, MI, USA) was poured onto the mold and degassed to vacuum for 30.0 min. After curing at 75.0°C for 2 h in an oven, the PDMS was peeled off carefully and cut into the designed shape. 20.0-μm thick PDMS membrane was produced by spin-coating PDMS prepolymer (10:1) on a plastic wafer with 3500 rpm for 60.0 s, and then was baked at 65.0°C for 30.0 min. Then, the bottom chip was sealed with PDMS membrane via oxygen plasma. Finally, the top replica were aligned carefully with bottom PDMS that containing membrane via oxygen plasma.

### The Clark electrode for measuring dissolved oxygen in cell medium

The determination of oxygen content in cell medium was conducted by electrochemical sensors based on Clark oxygen electrode^34^,^35^. Three kinds of microchips were fabricated for cell-culturing with the same design except a variation of distance between main channel and chambers. The distance was 1.50 mm, 2.00 mm, and 3.00 mm, respectively. These three kinds of microchips can be used to detect dissolved oxygen content relative to different chamber-channel distances. Before the detection process started, the oxygen of cell medium was removed by air blowing of nitrogen. Then, the up-layer channel was filled out with different content oxygen gas, and 10.0 mL treated cell medium was non-stop introduced to the bottom-layer channels. The cell medium was finally collected into a closed-centrifuge tube. After that, the dissolved oxygen of collected cell medium was detected by Clark-type oxygen electrodes according to the manufacturer's instructions of Horiba U-50 (Kyoto, Japan).

### Aptamer based microfluidic device for VEGF_165_ detection

In order to coat the aptamer in the detection regions of microchannel, outlets of cell culture channels were blocked by plugs to stop the liquid from flowing into other region. Then, the microchannels were modified by the injection of specific aptamer reagent. The procedure of coating aptamer on the microchannel was processed according to a developed method which has been described in previous work^3^. Firstly, channels full of carboxylatedsilancance were put into a vacuum environment for 30 min. After that, the microchannels were washed with PBS buffer (pH 7.4) for three times. Secondly, the carboxylic group was activated by standard amine coupling procedure using freshly prepared mixture solution of NHS solution (0.20 M) and EDC solution (0.80 M). Thirdly, the amino-modified aptamer with 2% BSA was injected into the microchannel for immobilization. The unbound aptamer was removed by washing the channels with 0.010 M PBS buffer containing 0.1% Tween-20 (PBST) for three times. After that, PBST was introduced to block the blank sites, and the device was stored at 4°C for further use. For VEGF detection, a fresh solution with ABTS (1.80 mM) and H_2_O_2_ substrate (2.92 mM) was added in each channel after the injection of aptamer and 100.0 μM hemin. Detection aptamer, a functional nucleic acid sequence containing aptamer and G-quadruplex was used to detect VEGF. At last, the green-blue color or absorbance produced by G-quadruplex DNAzyme-hemin-ABTS-peroxide was characterized using naked eye or quantified by UV−vis absorption spectra. The UV−vis absorption spectra detection photos were obtained by microscope equipped with a CCD camera after 10.0 min reaction. In order to ensure the reliability and accuracy of this method, VEGF_165_ was also detected by the commercial ELISA kit.

### Cells co-culture in the microchip under oxygen condition

CaSki cells and HUVECs were cultured in RPMI 1640 media (GIBCO, Grand Island, NY) with 10% fetal bovine serum (FBS), 100.0 μg/mL penicillin, and 100.0 μg/mL streptomycin. Prior to culturing CaSki and HUVEC cells on chip, the microchannels were coated with 2.0% FBS at 4°C overnight and then flushed by water. After that, the microchip was exposed under UV light for 0.50 h. Caski cells and HUVECs with a concentration of 10^6^ cells/mL were injected into central channel and middle channel with chambers, respectively. After culturing for 6.0 h, the cells adhered to the PDMS membrane. The initial medium was replaced by a new medium including only 2% fetal bovine serum (FBS) to decrease the discrepancy. Then, the microchip was overturned. At last, the mixture gas was applied to top layer of the microchip to make a hypoxia microenvironment.

### Characterization of cells

CCK-8 kit was utilized to monitor the cell proliferation under hypoxia treatment. Cell apoptosis was characterized by Hoechst 33342. Hoechst 33342 (100.0 μM) was incubated with these two types of cells for 20.0 min at 37°C and the images were collected by fluorescence microscopy (Leica DMI 4000 B, Germany). Cell viabilities of CaSki cells and HUVECs were determined by calcein AM and ethidiumhomodimer (live/dead kit).

### RNA Extraction and DNA amplification by PCR

RNA was extracted by EZNA Total RNA kit (Omega Bio-Tek; Doraville, GA) according to the manufacturer's instructions after the two kinds of cells were collected from cell microchannels after trypsin digestion. The kits from TiangenBiotech were employed for cDNA transcription. Using the GAPDH as control gene, PCR process was used to study transcription level of HIF-1α, VEGF_165_ gene. The primers for HIF-1α, VEGF_165_ and GAPDH were listed in Table S1.

## Supplementary Material

Supplementary Information Supplementary Information

Supplementary Information Supplementary Video 1

Supplementary Information Supplementary Video 2

Supplementary Information Supplementary Video 3
