# Supplementary material for: Oxygen-induced cell migration and on-line monitoring biomarkers modulation of cervical cancers on a microfluidic system
Source: Sci Rep. 2015 Apr 23;5:9643. doi: 10.1038/srep09643 (PMC5386116; doi:10.1038/srep09643)
Supplement: Supplementary Information [file srep09643-s1.pdf]

Supplementary Information

Oxygen-induced cell migration and on-line monitoring biomarkers modulation of cervical cancers on a microfluidic system

Xuexia Lin[1,3], Qiushui Chen[1,2], Wu Liu[1], Jie Zhang[1], Shiqi Wang[1],Zhixiong Lin[4]& Jin-Ming Lin[1,2,*]

[1]Beijing Key Laboratory of Microanalytical Methods and Instrumentation, Department of Chemistry, Tsinghua University, Beijing 100084, China

[2]The Key Laboratory of Bioorganic Phosphorus Chemistry & Chemical Biology, Department of Chemistry, Tsinghua University, Beijing 100084, China

[3]School of Science, Beijing University of Chemical Technology, Beijing 100029, China

[4]Department of Neurosurgery, First Affiliated Hospital of Fujian Medical University, Fuzhou, Fujian 350005, China

*Correspondence should be addressed to J.M. L (jmlin@mail.tsinghua.edu.cn)

EXPERIMENTAL

Reagents

2,2'-Azinobis(3-ethylbenzothiozoline)-6-sulfonic acid (ABTS), hemin, phosphate buffer saline (PBS), concanavalin A and human thrombin were purchased from Sigma Aldrich Chemical Co. (St. Louis, MO., USA). Bovine serum albumin (BSA), human immunoglobulin G (IgG) and L-ascorbic acid were obtained from Dingguo Biotechnology Co., Ltd. (Beijing, China). Fetal bovine serum (FBS) was purchased from hyclone (Logan, UT). SYBR Gold prepared with TE buffer (10 mMTris–HCl, pH 8.0, 1 mM EDTA) was purchased from Invitrogen Corporation (Karlsruhe, Germany). A Live/Dead assay kit (Calcein-AM/EthD-1)

was gotten from Invitrogen (CA, USA). Cell apoptosis was detected by Hoechst 33342 (Invitrogen, CA, USA).Generation of ROS was analyzed by dihydroethidium (DHE, Beijing, China).

RESULTS AND DISCUSSION

Fabrication of Microfluidic Device

A two-layer microfluidic device was designed with three kinds of channels in this work. Chambers away from the central channel were 1.50 mm, 2.00 mm, 3.00 mm, respectively. Width of the narrow channels was designed as 0.10 mm to mimic the surrounding environment of cells (Fig.S1A). The depth of the cell culture channels and the chamber were 82.0 μm, and the connection channels depth was 24.0μm (Fig.S1D and F). Altitude differences between cell culture channels and narrow channels were designed to control the cell growth environment. As shown in Fig. S2, the narrow channels integrated on the microfluidic device could stop the cells from leaking into next channels and promote the exchange of material. The chip design ensured no cells cross-interference and kept the substance exchanging between two kinds of cell lines. Moreover, due to the elastic characteristic of PDMS, micro-columns in this composition were served as microvalves to control the fluidic direction in middle channels. These PDMS micro-columns were away from cell chambers about 1.50 mm and the interspace between micro-columns was 50.0 μm (Fig.S1B). The chamber would be isolated when the pressure was applied on two parts of adjacent micro-columns and the chamber turned to open again when the pressure was released (Supplementary Video 1). Supplementary Video 2 demonstrated that the utilizing of channels

and microvalves could guarantee the determination of cells secretion in each chamber without cross-contamination. In order to mimic the oxygen gradient microenvironment, a two-layer microchip was applied, and 20.0-μm thickness PDMS membrane was exploited (Fig. S1C). Due to the excellent gas permreability, PDMS membrane was employed in reducing the shear-stress produced by mixture gas under different oxygen conditions. In addition, this PDMS membrane was enabled to decrease the cell medium volatility under different oxygen condition. The gas pressure was detected by nitrogen blowing instrument with gas measuring flowmeter. As shown in Fig. S3, the pressure of inlet changed little due to gas diffusion. Compared with the inlet pressure, the outlet pressure was changed slightly by applying PDMS membrane. These inconspicuous differences were ignored in the next experiments.

Determination of VEGF165 Based on Nucleic Acid Aptamer

For on-line cell secretion monitoring, the microfluidic device was modified with aptamer and used for determination of VEGF165 protein. SYBR Gold was used to investigate whether aptamer and functional nucleic acid were effective for VEGF165 protein detection. As shown in Fig. S5, the target VEGF165 protein could be captured by the aptamer successfully and detected by functional nucleic acid consisting of aptamer and G-quadruplex structure. A high specificity of VEGF165 detection was obtained by the using of functional nucleic acid based system (Fig. S6A). The linearity of VEGF165 from cell medium ranged from 1.50 ng/mL to 36.0 ng/mL was achieved by UV-Vis absorption (Fig. S6B). Because of the relative high background resulting from the G-quadruplex structure of VEGF165 protein aptamer, the limit of detection was up to 1.30 ng/mL with a signal to noise ratio of 3.0. These results implied

that the microchip integrated with aptamer-based microchannels was feasible for VEGF165 4 assay. Moreover, G-quadruplex catalyzed ABTS–hemin–H_2O_2 system produced the green-blue color1 which could be considered to be an auxiliary detection method. Therefore, VEGF165 protein can be semi-quantitatively analyzed by naked eyes. Fig. S6C showed that green colors were generated when the concentration of the VEGF165 protein ranged from 5.00 ng/mL to 36.0 ng/mL. The photographs were taken by microscope with CCD camera.

REFERENCES

1. Lin, X.; Chen, Q.; Liu, W.; Li, H.; Lin, J.-M. *Biosens. Bioelectron.***2014**, *56*, 71.

Table S1. Primers of HIF-1α and $VEGF_{165}$ genes for PCR.

Name	Primer Sequence(5'-3')	Length (bp)
HIF-1α	F:AACAAAAACACAGCGAAGC<br>R:ATAGTGAATGTGGCCTGTG	124
$VEGF_{165}$	F: AGGGCAGAACATCACGAAG<br>R:ACTCCAGGCCCTCGTCATTG	182
GAPDH	F:AACTTTGGCATTGTGGAAGGGCTC<br>R:ACCCTGTTGCTGTAGCCGTATTCA	275

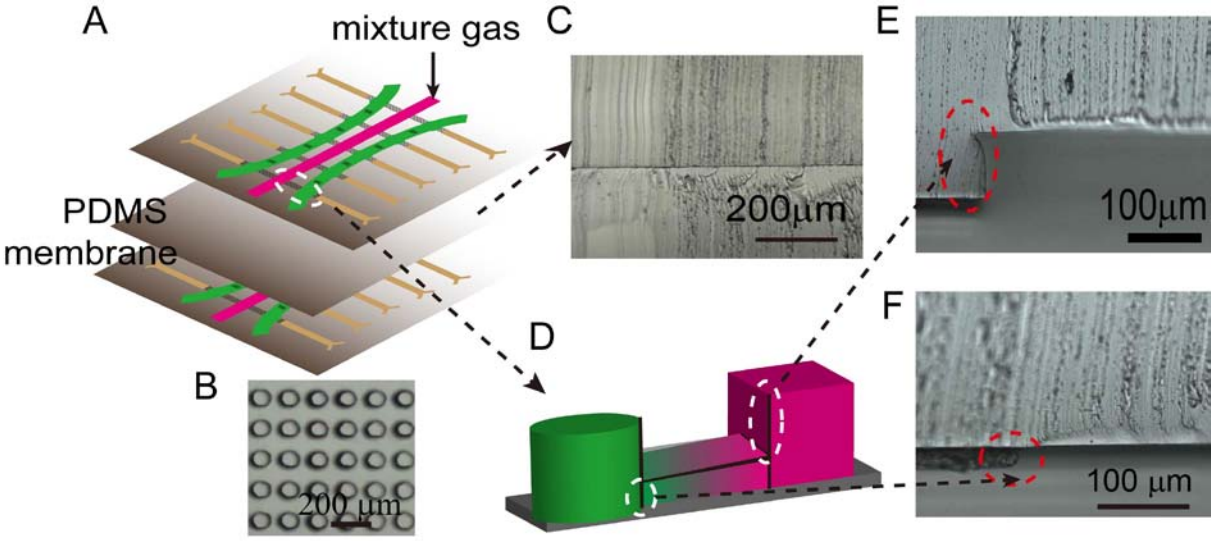

Figure S1. Fabrication of two-lay microfluidic device. (A) Two–lay microchip with PDMS membrane. (B) The microcolumns as microvalve. (C)An actual of PDMS membrane. (D) Altitude difference of the microchannels. (E) The depths of cell culture channels and the connection channels. (F) The depth of connection channels.

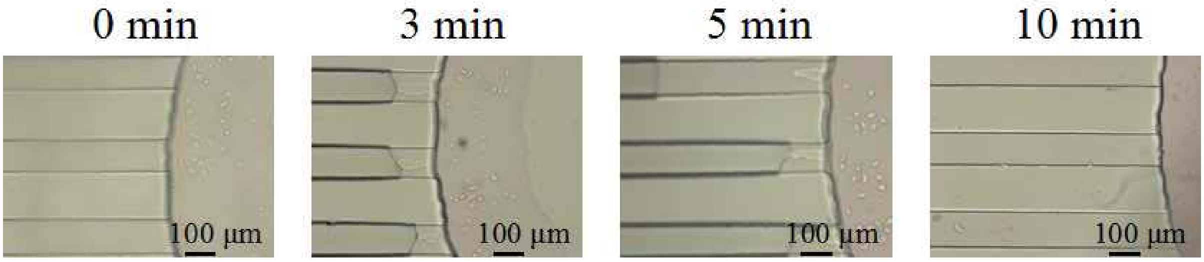

Figure S2. The images cell medium diffusion into connection channels. Experiments were repeated three times in parallel.

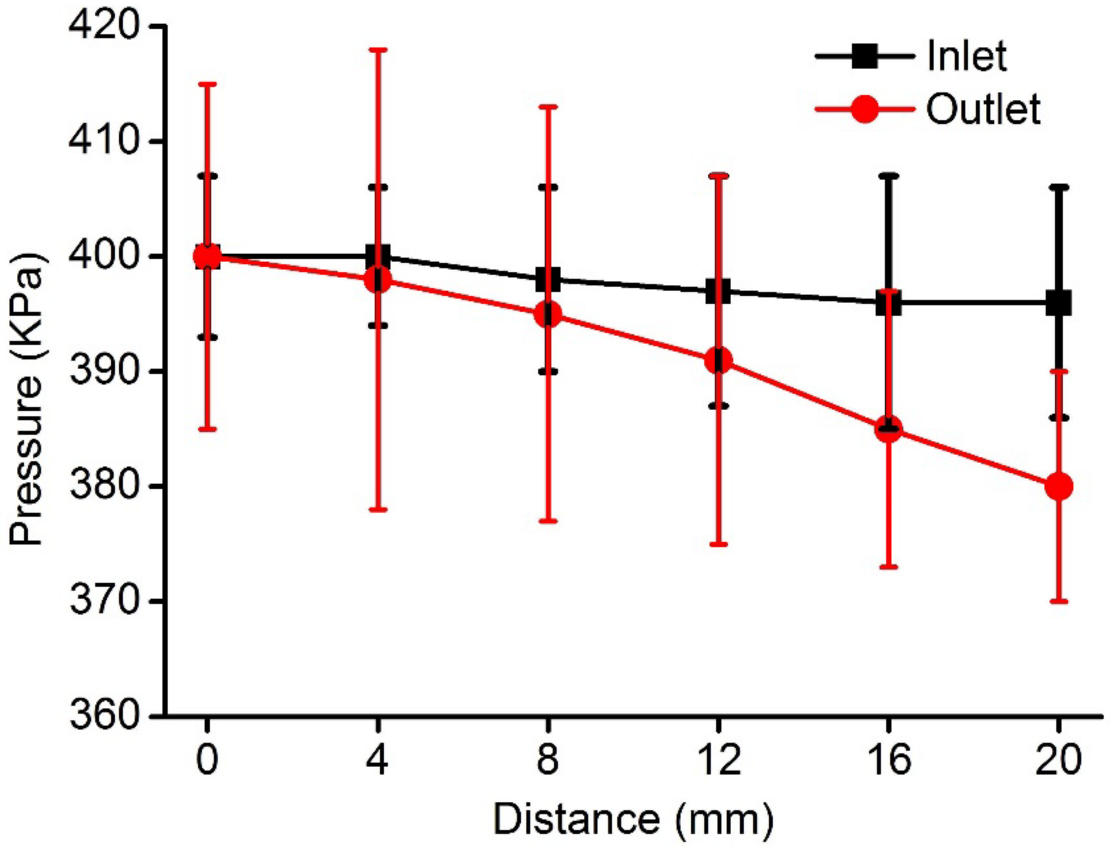

Figure S3. The alteration of pressure with PDMS membrane. The thick of PDMS membrane was 20 µm.

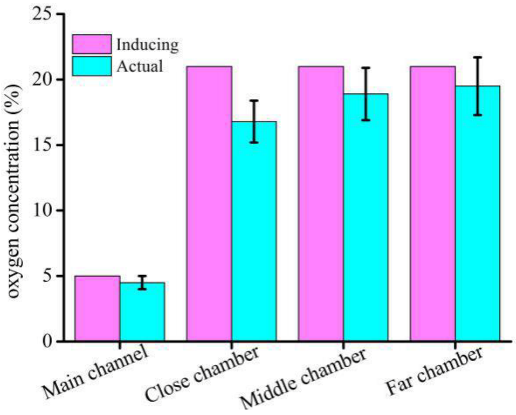

Figure S4. Evaluation of oxygen content in cell medium by iodine method. The concentration of oxygen by inducing 5% O_2 to main channel, and 21% O_2 to both sides channels.

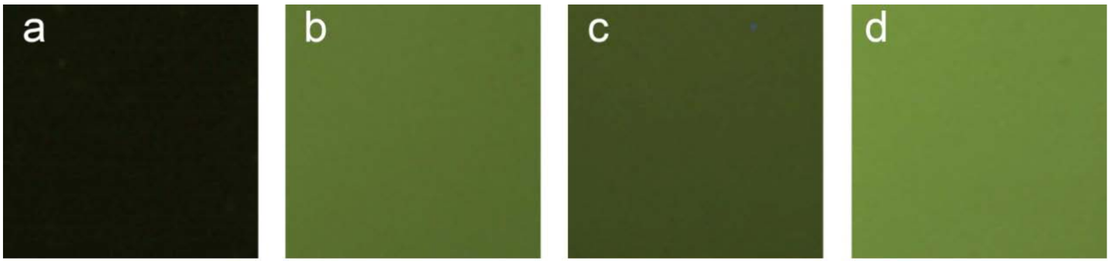

Figure S5. The fluorescence images of aptamer for $VEGF_{165}$ assay by SYBR Gold dye labelling. (a) Blank; (b) microchannels coating aptamer; (c) the $VEGF_{165}$ protein captured by aptamer; (d) the determination of $VEGF_{165}$ by functional nucleic acid.

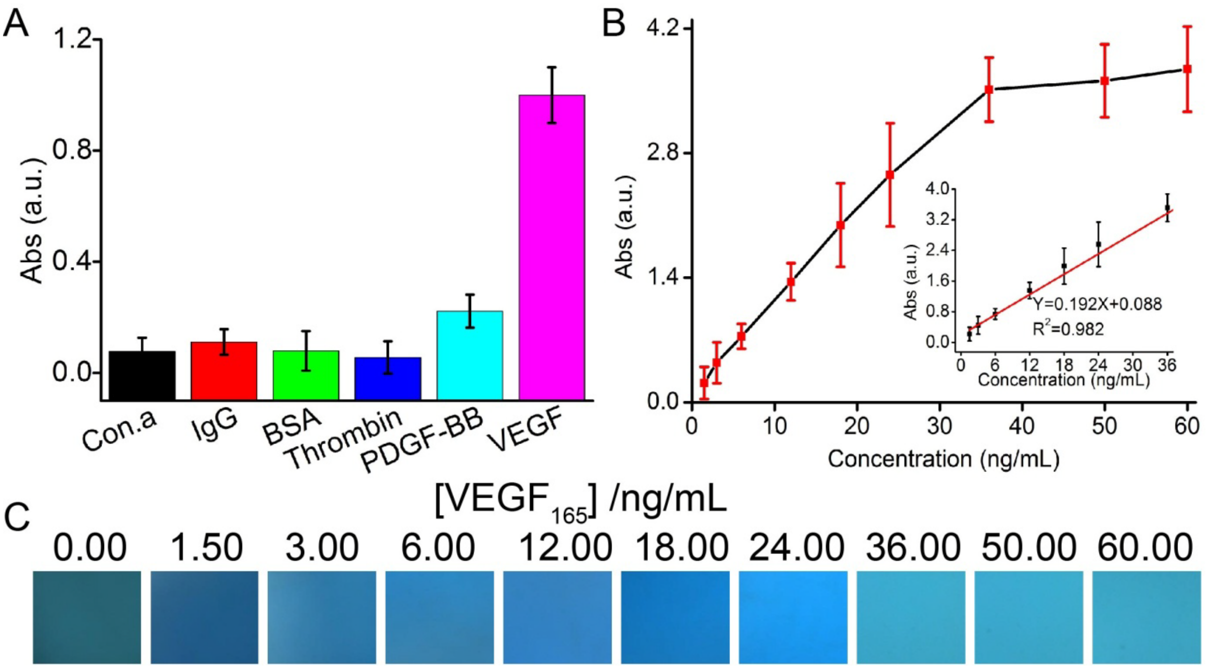

Figure S6. Determination of $VEGF_{165}$ protein by aptamer-based microchip. (A) Specificity of the functional nucleic acid based VEGF $_{165}$ assay. The concentrations of proteins were 12.00 ng/mL. (B) Absorbance of the aptasensing microfluidic chip with different concentrations of $VEGF_{165}$ (from 1.50 ng/mL to 60.0 ng/mL). (C) The responding color graph for different concentrations of $VEGF_{165}$.

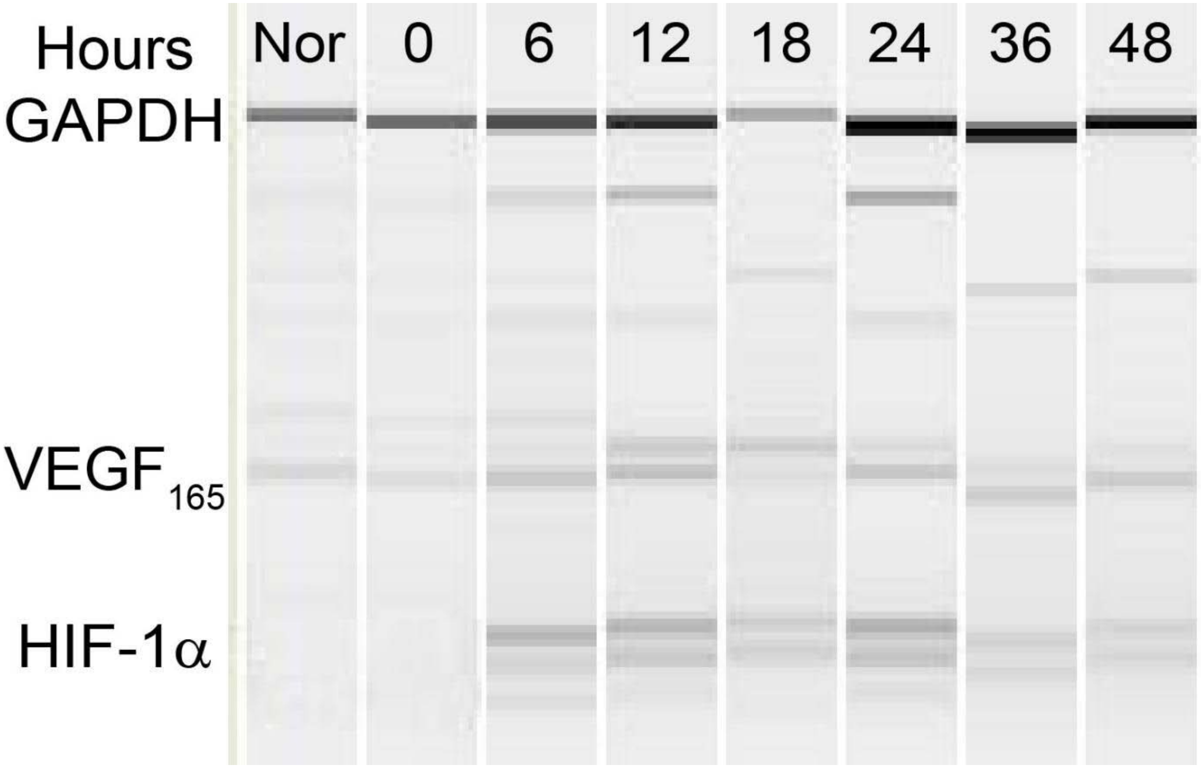

Figure S7. Time-dependence HIF-1α and $VEGF_{165}$ genes for HUVECs by 2% hydroxyethyl cellulose electrophoresis. The control gene was GAPDH. Normoxia indicated that Cells culture with 21% O_2, the other cells cultured at 5% $O_{2.}$

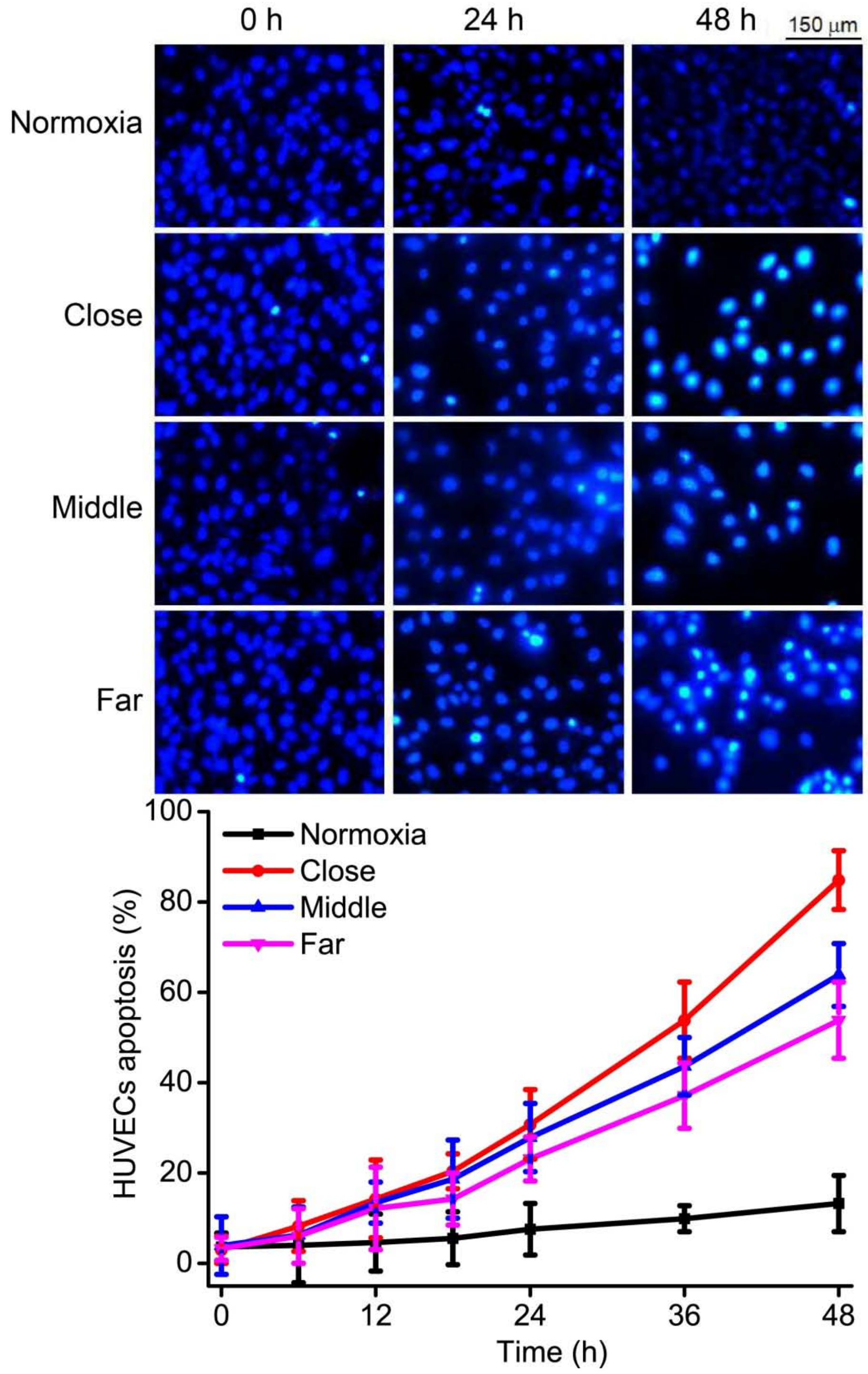

Figure S8. Fluorescent images of HUVECs apoptosis in different chambers under co-culture with 5% O_2 condition. Normoxia indicated that Cells culture with 21% O_2, the other cells cultured at 5% O_2. Cells were labeled by Hoechst 33342.

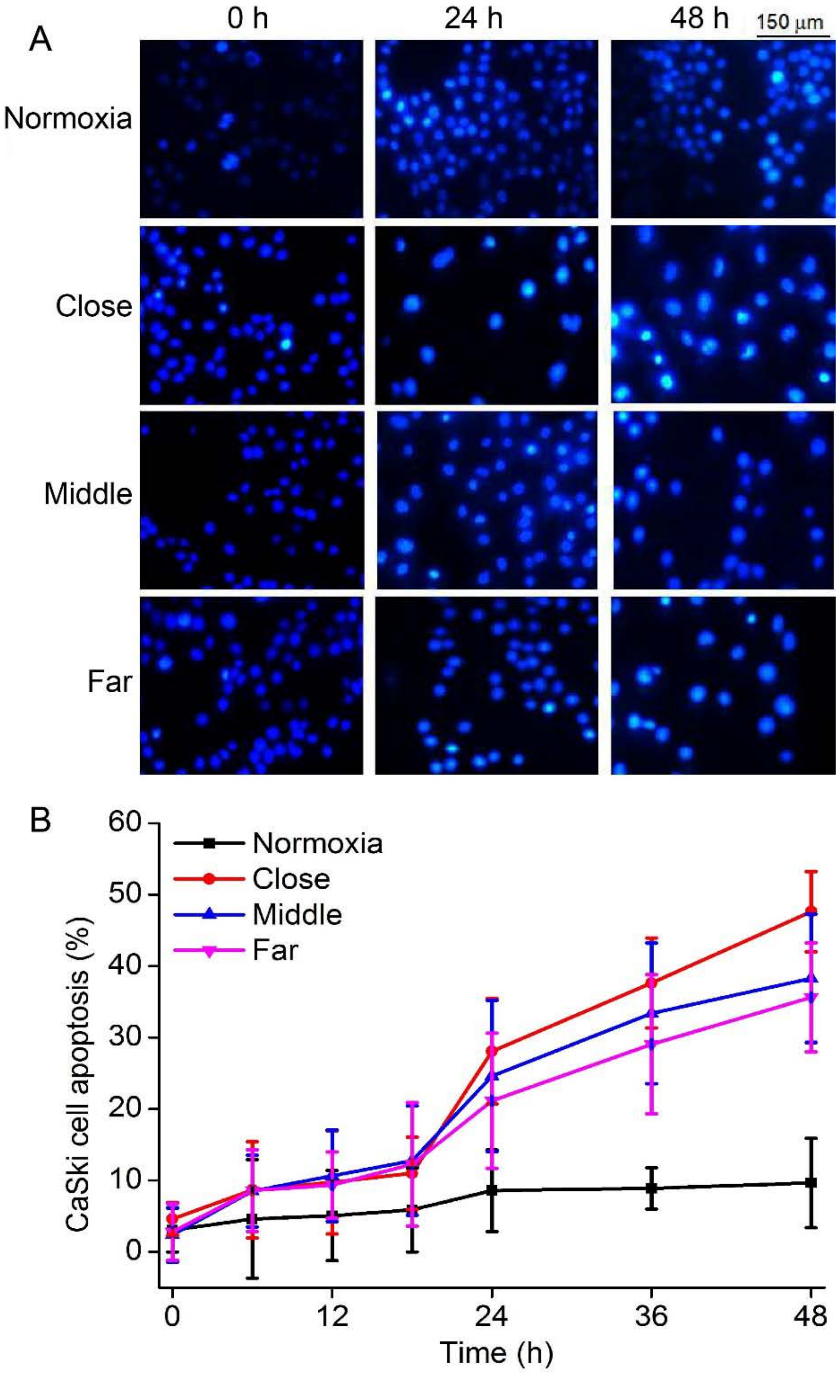

Figure S9. (A) Fluorescent images of CaSki cells apoptosis in different chambers under co-culture with 5% O_2 condition. (B) Apoptosis of CaSki cells cultured in different chambers during 1–2 days. Normoxia indicated that Cells culture with 21% O_2, the other cells cultured at 5% O_2. Cells were labeled by Hoechst 33342.

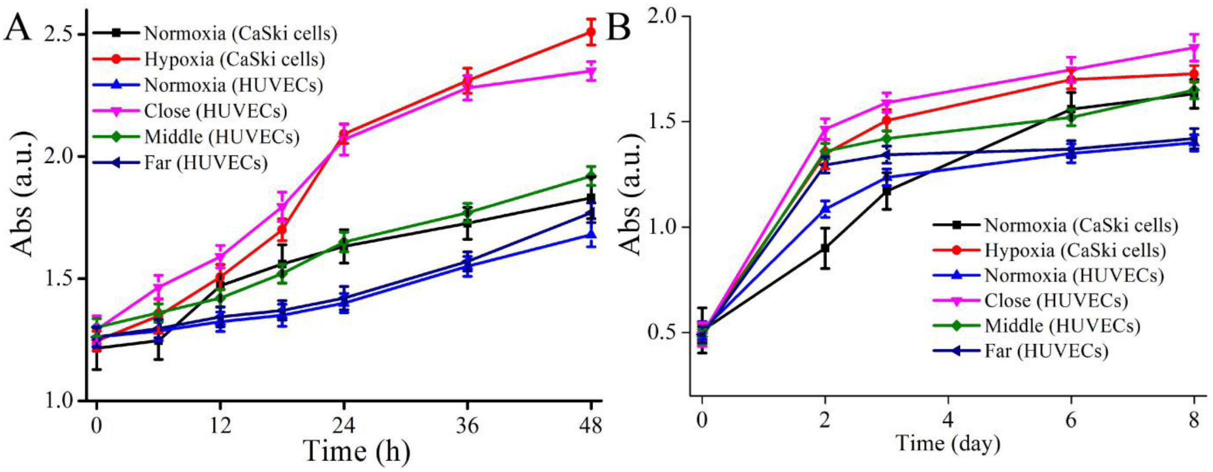

Figure S10. The absorbance of CCK-8 for CaSki cells proliferation. Control indicated that cells culture with 21% O_2. During 48 h, the other cells cultured at 5% $O_{2.}$ During 8 days, the other cells cultured at 15% $O_{2.}$
